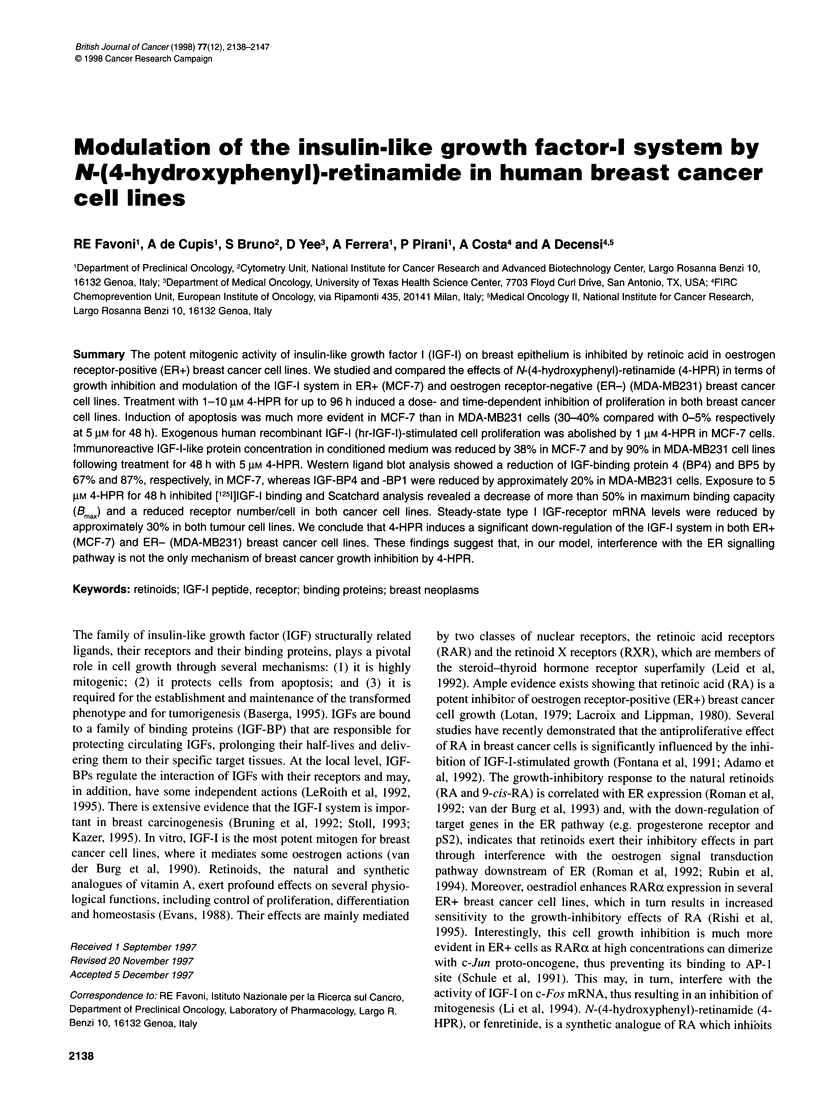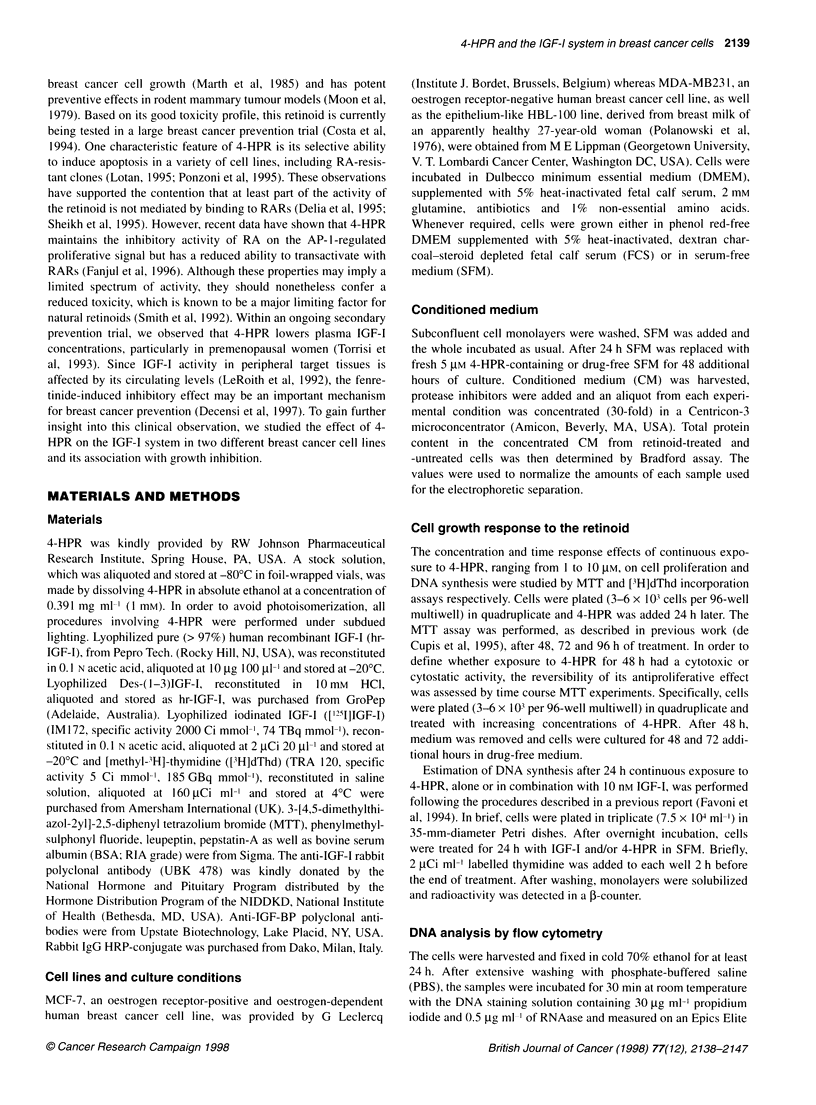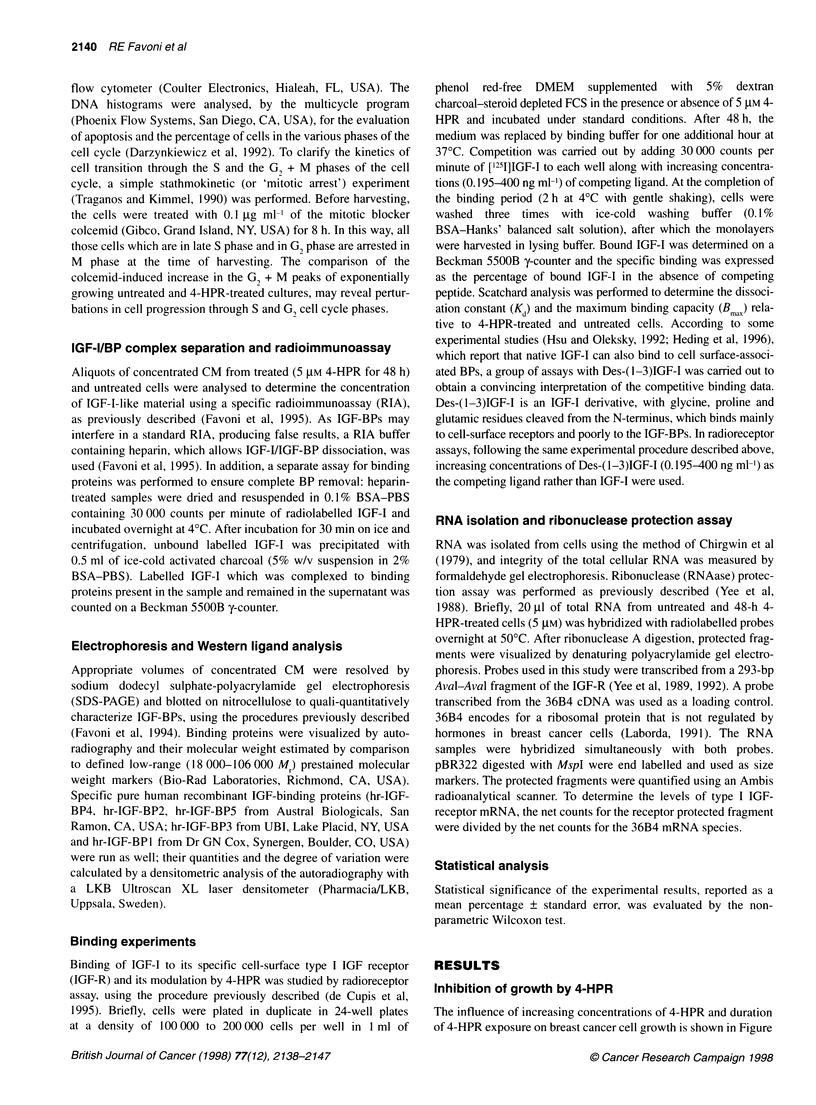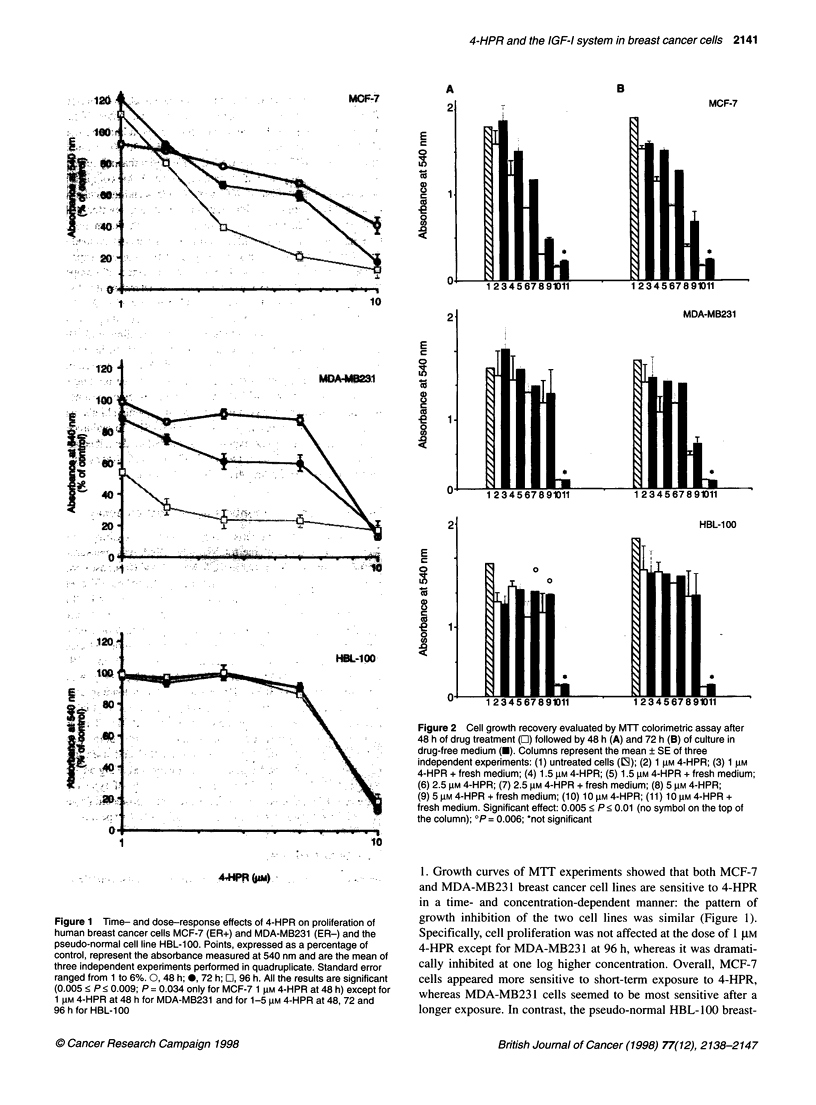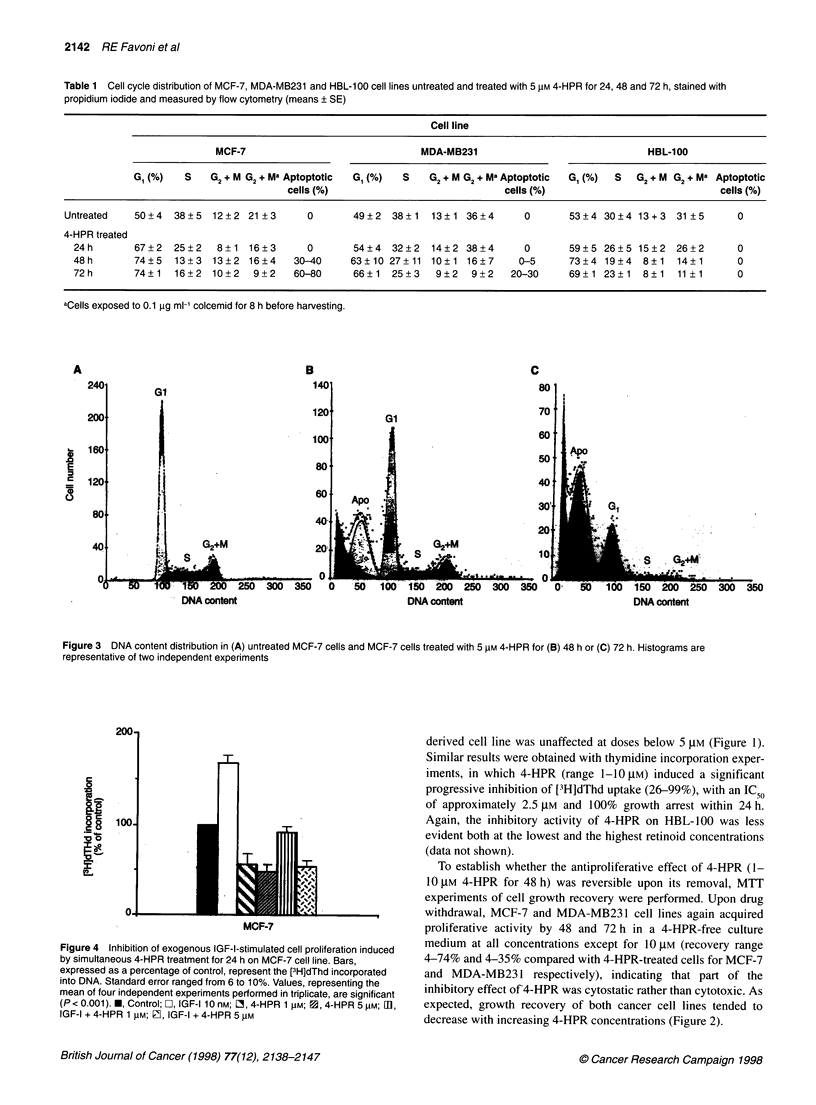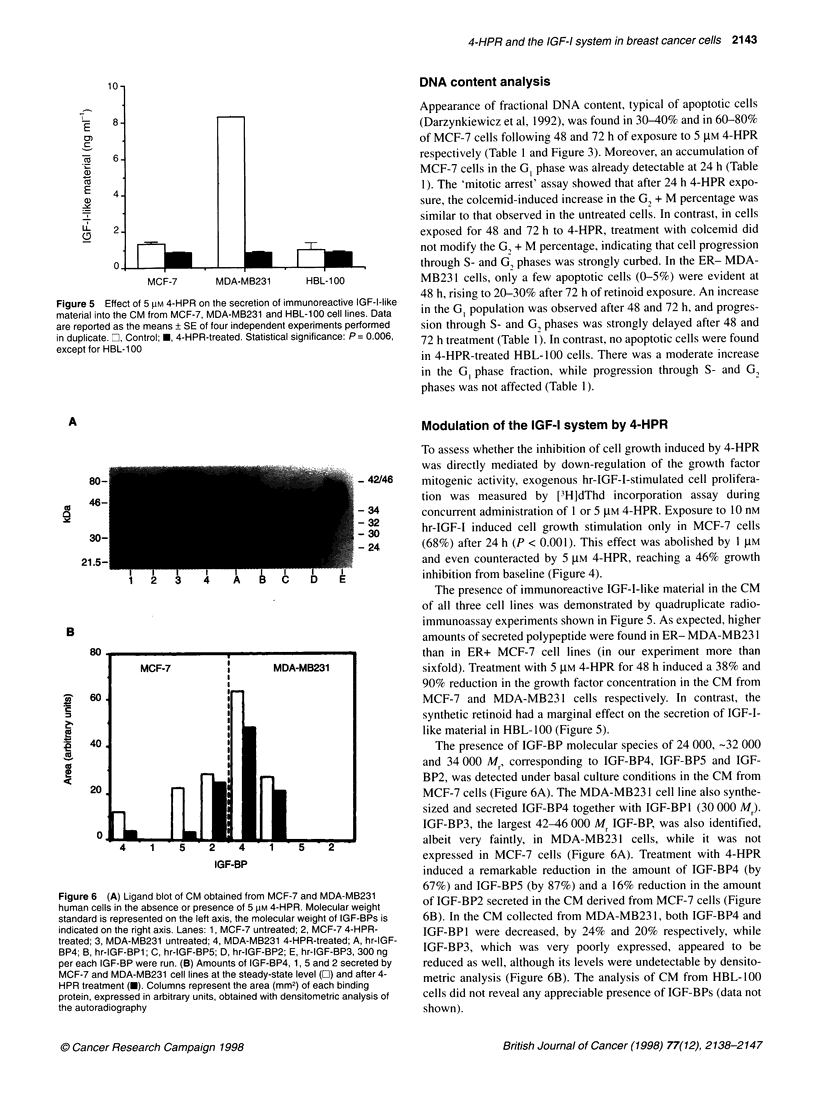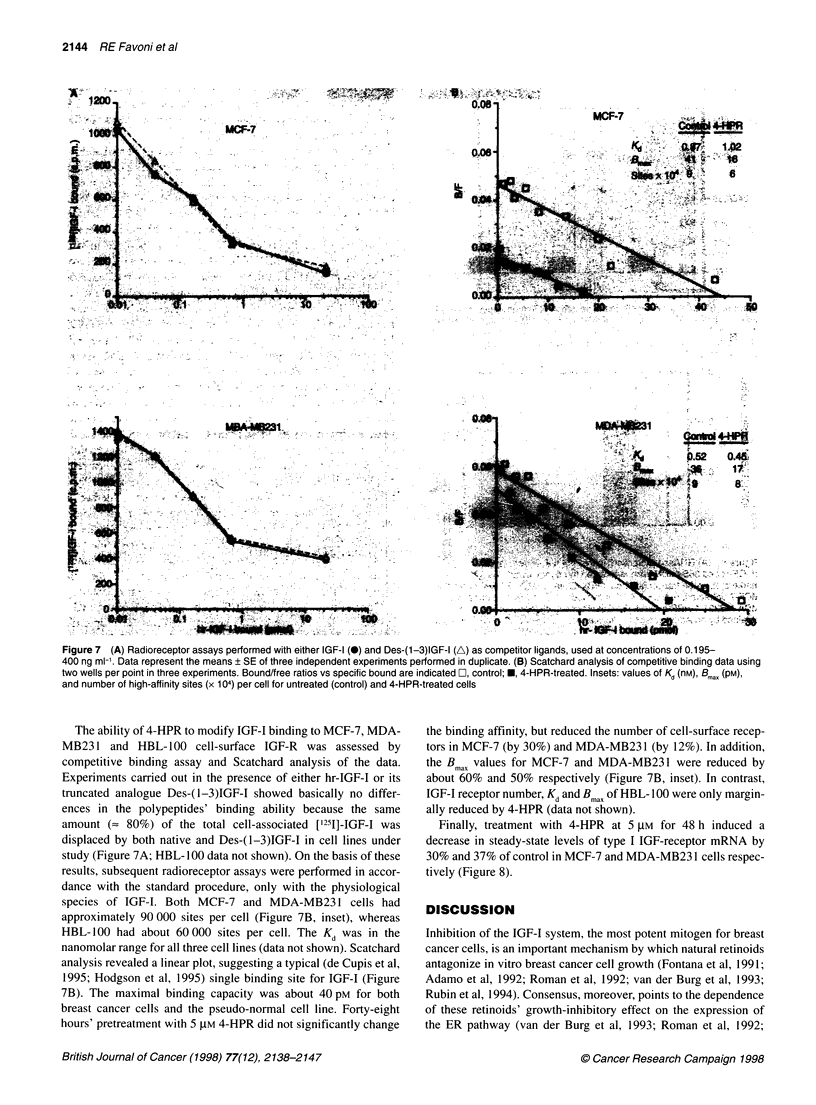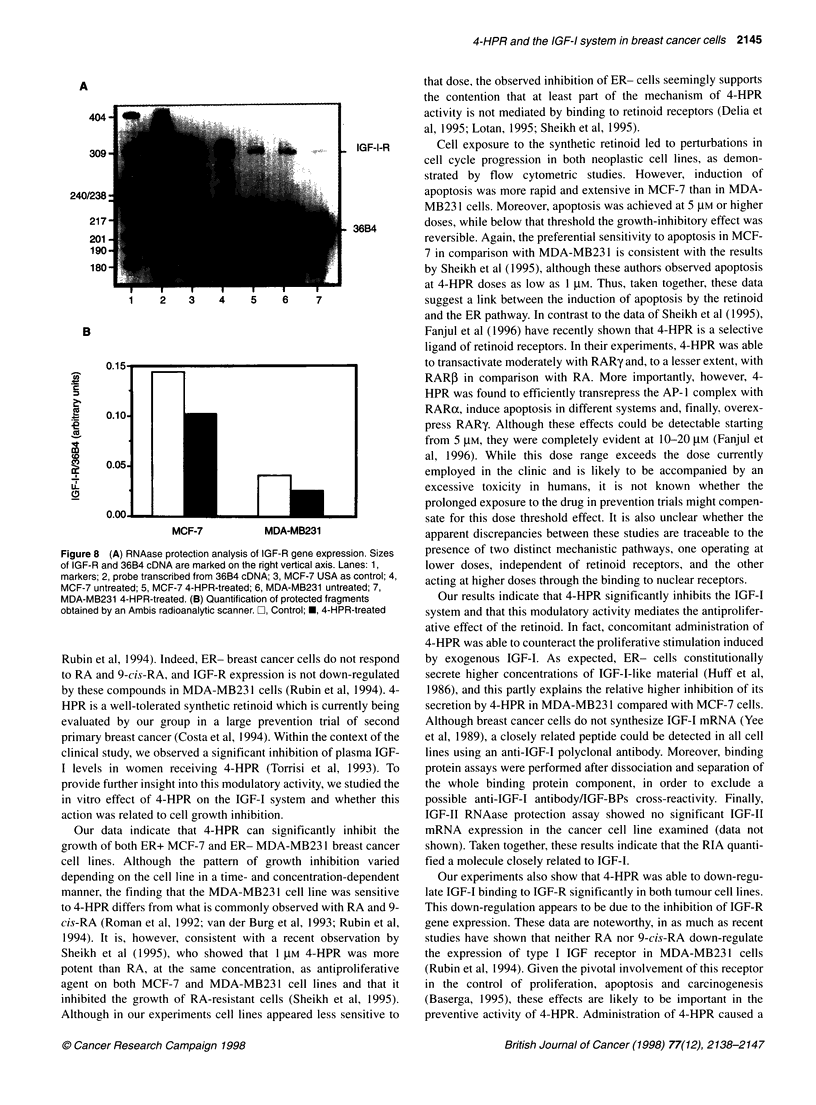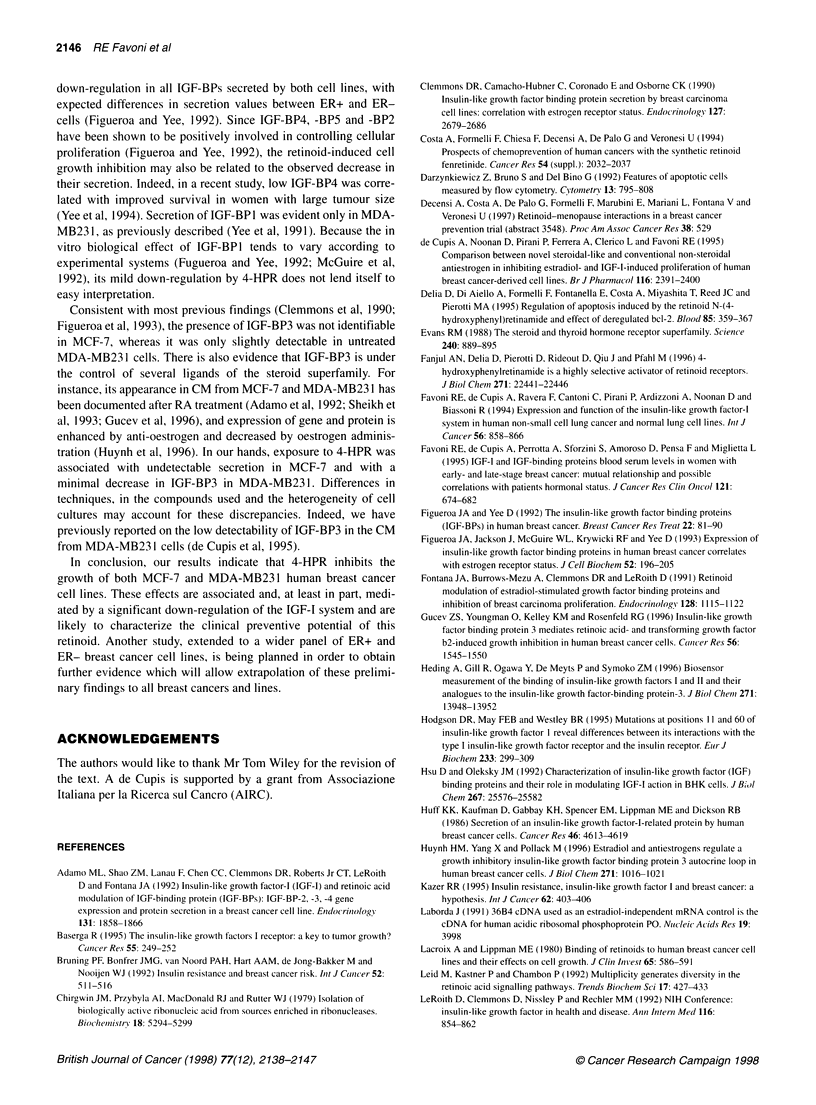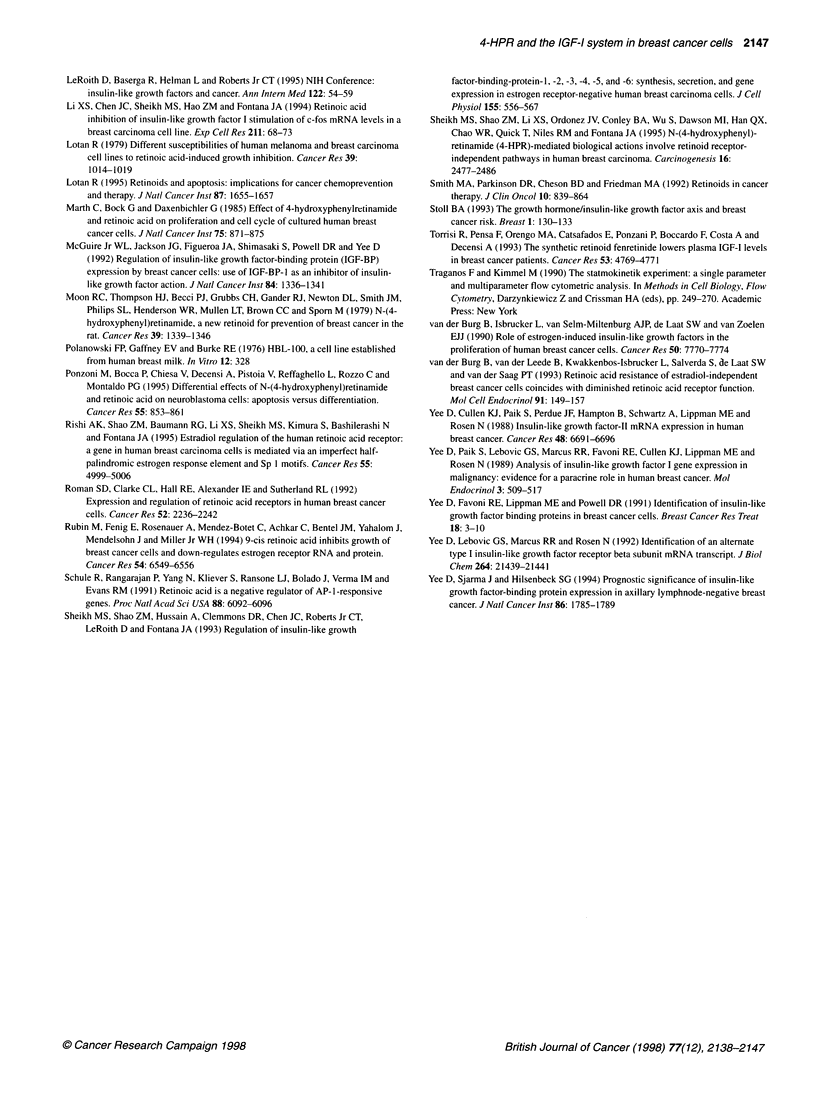# Modulation of the insulin-like growth factor-I system by N-(4-hydroxyphenyl)-retinamide in human breast cancer cell lines.

**DOI:** 10.1038/bjc.1998.358

**Published:** 1998-06

**Authors:** R. E. Favoni, A. de Cupis, S. Bruno, D. Yee, A. Ferrera, P. Pirani, A. Costa, A. Decensi

**Affiliations:** Department of Preclinical Oncology, National Institute for Cancer Research and Advanced Biotechnology Center, Genoa, Italy.

## Abstract

**Images:**


British Journal of Cancer (1998) 77(12), 2138–2147
© 1998 Cancer Research Campaign

### Modulation of the insulin-like growth factor-I system by *N*-(4-hydroxyphenyl)-retinamide in human breast cancer cell lines

**RE Favoni[1], A de Cupis[1], S Bruno[2], D Yee[3], A Ferrera[1], P Pirani[1], A Costa[4] and A Decensi[4,5]**

[1]Department of Preclinical Oncology, [2]Cytometry Unit, National Institute for Cancer Research and Advanced Biotechnology Center, Largo Rosanna Benzi 10, 16132 Genoa, Italy; [3]Department of Medical Oncology, University of Texas Health Science Center, 7703 Floyd Curl Drive, San Antonio, TX, USA; [4]FIRC Chemoprevention Unit, European Institute of Oncology, via Ripamonti 435, 20141 Milan, Italy; [5]Medical Oncology II, National Institute for Cancer Research, Largo Rosanna Benzi 10, 16132 Genoa, Italy

**Summary** The potent mitogenic activity of insulin-like growth factor I (IGF-I) on breast epithelium is inhibited by retinoic acid in oestrogen receptor-positive (ER+) breast cancer cell lines. We studied and compared the effects of *N*-(4-hydroxyphenyl)-retinamide (4-HPR) in terms of growth inhibition and modulation of the IGF-I system in ER+ (MCF-7) and oestrogen receptor-negative (ER–) (MDA-MB231) breast cancer cell lines. Treatment with 1–10 μM 4-HPR for up to 96 h induced a dose- and time-dependent inhibition of proliferation in both breast cancer cell lines. Induction of apoptosis was much more evident in MCF-7 than in MDA-MB231 cells (30–40% compared with 0–5% respectively at 5 μM for 48 h). Exogenous human recombinant IGF-I (hr-IGF-I)-stimulated cell proliferation was abolished by 1 μM 4-HPR in MCF-7 cells. Immunoreactive IGF-I-like protein concentration in conditioned medium was reduced by 38% in MCF-7 and by 90% in MDA-MB231 cell lines following treatment for 48 h with 5 μM 4-HPR. Western ligand blot analysis showed a reduction of IGF-binding protein 4 (BP4) and BP5 by 67% and 87%, respectively, in MCF-7, whereas IGF-BP4 and -BP1 were reduced by approximately 20% in MDA-MB231 cells. Exposure to 5 μM 4-HPR for 48 h inhibited [$^{125}$I]IGF-I binding and Scatchard analysis revealed a decrease of more than 50% in maximum binding capacity ($B_{max}$) and a reduced receptor number/cell in both cancer cell lines. Steady-state type I IGF-receptor mRNA levels were reduced by approximately 30% in both tumour cell lines. We conclude that 4-HPR induces a significant down-regulation of the IGF-I system in both ER+ (MCF-7) and ER– (MDA-MB231) breast cancer cell lines. These findings suggest that, in our model, interference with the ER signalling pathway is not the only mechanism of breast cancer growth inhibition by 4-HPR.

**Keywords:** retinoids; IGF-I peptide, receptor; binding proteins; breast neoplasms

The family of insulin-like growth factor (IGF) structurally related ligands, their receptors and their binding proteins, plays a pivotal role in cell growth through several mechanisms: (1) it is highly mitogenic; (2) it protects cells from apoptosis; and (3) it is required for the establishment and maintenance of the transformed phenotype and for tumorigenesis (Baserga, 1995). IGFs are bound to a family of binding proteins (IGF-BP) that are responsible for protecting circulating IGFs, prolonging their half-lives and delivering them to their specific target tissues. At the local level, IGF-BPs regulate the interaction of IGFs with their receptors and may, in addition, have some independent actions (LeRoith et al, 1992, 1995). There is extensive evidence that the IGF-I system is important in breast carcinogenesis (Bruning et al, 1992; Stoll, 1993; Kazer, 1995). In vitro, IGF-I is the most potent mitogen for breast cancer cell lines, where it mediates some oestrogen actions (van der Burg et al, 1990). Retinoids, the natural and synthetic analogues of vitamin A, exert profound effects on several physiological functions, including control of proliferation, differentiation and homeostasis (Evans, 1988). Their effects are mainly mediated by two classes of nuclear receptors, the retinoic acid receptors (RAR) and the retinoid X receptors (RXR), which are members of the steroid–thyroid hormone receptor superfamily (Leid et al, 1992). Ample evidence exists showing that retinoic acid (RA) is a potent inhibitor of oestrogen receptor-positive (ER+) breast cancer cell growth (Lotan, 1979; Lacroix and Lippman, 1980). Several studies have recently demonstrated that the antiproliferative effect of RA in breast cancer cells is significantly influenced by the inhibition of IGF-I-stimulated growth (Fontana et al, 1991; Adamo et al, 1992). The growth-inhibitory response to the natural retinoids (RA and 9-*cis*-RA) is correlated with ER expression (Roman et al, 1992; van der Burg et al, 1993) and, with the down-regulation of target genes in the ER pathway (e.g. progesterone receptor and pS2), indicates that retinoids exert their inhibitory effects in part through interference with the oestrogen signal transduction pathway downstream of ER (Roman et al, 1992; Rubin et al, 1994). Moreover, oestradiol enhances RARα expression in several ER+ breast cancer cell lines, which in turn results in increased sensitivity to the growth-inhibitory effects of RA (Rishi et al, 1995). Interestingly, this cell growth inhibition is much more evident in ER+ cells as RARα at high concentrations can dimerize with c-*Jun* proto-oncogene, thus preventing its binding to AP-1 site (Schule et al, 1991). This may, in turn, interfere with the activity of IGF-I on c-*Fos* mRNA, thus resulting in an inhibition of mitogenesis (Li et al, 1994). *N*-(4-hydroxyphenyl)-retinamide (4-HPR), or fenretinide, is a synthetic analogue of RA which inhibits

*Received 1 September 1997*
*Revised 20 November 1997*
*Accepted 5 December 1997*

*Correspondence to:* RE Favoni, Istituto Nazionale per la Ricerca sul Cancro, Department of Preclinical Oncology, Laboratory of Pharmacology, Largo R. Benzi 10, 16132 Genoa, Italy breast cancer cell growth (Marth et al, 1985) and has potent preventive effects in rodent mammary tumour models (Moon et al, 1979). Based on its good toxicity profile, this retinoid is currently being tested in a large breast cancer prevention trial (Costa et al, 1994). One characteristic feature of 4-HPR is its selective ability to induce apoptosis in a variety of cell lines, including RA-resistant clones (Lotan, 1995; Ponzoni et al, 1995). These observations have supported the contention that at least part of the activity of the retinoid is not mediated by binding to RARs (Delia et al, 1995; Sheikh et al, 1995). However, recent data have shown that 4-HPR maintains the inhibitory activity of RA on the AP-1-regulated proliferative signal but has a reduced ability to transactivate with RARs (Fanjul et al, 1996). Although these properties may imply a limited spectrum of activity, they should nonetheless confer a reduced toxicity, which is known to be a major limiting factor for natural retinoids (Smith et al, 1992). Within an ongoing secondary prevention trial, we observed that 4-HPR lowers plasma IGF-I concentrations, particularly in premenopausal women (Torrisi et al, 1993). Since IGF-I activity in peripheral target tissues is affected by its circulating levels (LeRoith et al, 1992), the fenretinide-induced inhibitory effect may be an important mechanism for breast cancer prevention (Decensi et al, 1997). To gain further insight into this clinical observation, we studied the effect of 4-HPR on the IGF-I system in two different breast cancer cell lines and its association with growth inhibition.

### MATERIALS AND METHODS

### Materials

4-HPR was kindly provided by RW Johnson Pharmaceutical Research Institute, Spring House, PA, USA. A stock solution, which was aliquoted and stored at –80°C in foil-wrapped vials, was made by dissolving 4-HPR in absolute ethanol at a concentration of 0.391 mg $ml^{-1}$ (1 mM). In order to avoid photoisomerization, all procedures involving 4-HPR were performed under subdued lighting. Lyophilized pure (> 97%) human recombinant IGF-I (hr-IGF-I), from Pepro Tech. (Rocky Hill, NJ, USA), was reconstituted in 0.1 N acetic acid, aliquoted at 10 μg 100 $\mu l^{-1}$ and stored at –20°C. Lyophilized Des-(1–3)IGF-I, reconstituted in 10 mM HCl, aliquoted and stored as hr-IGF-I, was purchased from GroPep (Adelaide, Australia). Lyophilized iodinated IGF-I ($[^{125}I]$IGF-I) (IM172, specific activity 2000 Ci $mmol^{-1}$, 74 TBq $mmol^{-1}$), reconstituted in 0.1 N acetic acid, aliquoted at 2 μCi 20 $\mu l^{-1}$ and stored at –20°C and [methyl-$^3$H]-thymidine ($[^3H]$dThd) (TRA 120, specific activity 5 Ci $mmol^{-1}$, 185 GBq $mmol^{-1}$), reconstituted in saline solution, aliquoted at 160 μCi $ml^{-1}$ and stored at 4°C were purchased from Amersham International (UK). 3-[4,5-dimethylthiazol-2yl]-2,5-diphenyl tetrazolium bromide (MTT), phenylmethylsulphonyl fluoride, leupeptin, pepstatin-A as well as bovine serum albumin (BSA; RIA grade) were from Sigma. The anti-IGF-I rabbit polyclonal antibody (UBK 478) was kindly donated by the National Hormone and Pituitary Program distributed by the Hormone Distribution Program of the NIDDKD, National Institute of Health (Bethesda, MD, USA). Anti-IGF-BP polyclonal antibodies were from Upstate Biotechnology, Lake Placid, NY, USA. Rabbit IgG HRP-conjugate was purchased from Dako, Milan, Italy.

### Cell lines and culture conditions

MCF-7, an oestrogen receptor-positive and oestrogen-dependent human breast cancer cell line, was provided by G Leclercq (Institute J. Bordet, Brussels, Belgium) whereas MDA-MB231, an oestrogen receptor-negative human breast cancer cell line, as well as the epithelium-like HBL-100 line, derived from breast milk of an apparently healthy 27-year-old woman (Polanowski et al, 1976), were obtained from M E Lippman (Georgetown University, V. T. Lombardi Cancer Center, Washington DC, USA). Cells were incubated in Dulbecco minimum essential medium (DMEM), supplemented with 5% heat-inactivated fetal calf serum, 2 mM glutamine, antibiotics and 1% non-essential amino acids. Whenever required, cells were grown either in phenol red-free DMEM supplemented with 5% heat-inactivated, dextran charcoal–steroid depleted fetal calf serum (FCS) or in serum-free medium (SFM).

### Conditioned medium

Subconfluent cell monolayers were washed, SFM was added and the whole incubated as usual. After 24 h SFM was replaced with fresh 5 μM 4-HPR-containing or drug-free SFM for 48 additional hours of culture. Conditioned medium (CM) was harvested, protease inhibitors were added and an aliquot from each experimental condition was concentrated (30-fold) in a Centricon-3 microconcentrator (Amicon, Beverly, MA, USA). Total protein content in the concentrated CM from retinoid-treated and -untreated cells was then determined by Bradford assay. The values were used to normalize the amounts of each sample used for the electrophoretic separation.

### Cell growth response to the retinoid

The concentration and time response effects of continuous exposure to 4-HPR, ranging from 1 to 10 μM, on cell proliferation and DNA synthesis were studied by MTT and $[^3H]$dThd incorporation assays respectively. Cells were plated (3–6 × $10^3$ cells per 96-well multiwell) in quadruplicate and 4-HPR was added 24 h later. The MTT assay was performed, as described in previous work (de Cupis et al, 1995), after 48, 72 and 96 h of treatment. In order to define whether exposure to 4-HPR for 48 h had a cytotoxic or cytostatic activity, the reversibility of its antiproliferative effect was assessed by time course MTT experiments. Specifically, cells were plated (3–6 × $10^3$ per 96-well multiwell) in quadruplicate and treated with increasing concentrations of 4-HPR. After 48 h, medium was removed and cells were cultured for 48 and 72 additional hours in drug-free medium.

Estimation of DNA synthesis after 24 h continuous exposure to 4-HPR, alone or in combination with 10 nM IGF-I, was performed following the procedures described in a previous report (Favoni et al, 1994). In brief, cells were plated in triplicate (7.5 × $10^4$ $ml^{-1}$) in 35-mm-diameter Petri dishes. After overnight incubation, cells were treated for 24 h with IGF-I and/or 4-HPR in SFM. Briefly, 2 μCi $ml^{-1}$ labelled thymidine was added to each well 2 h before the end of treatment. After washing, monolayers were solubilized and radioactivity was detected in a β-counter.

### DNA analysis by flow cytometry

The cells were harvested and fixed in cold 70% ethanol for at least 24 h. After extensive washing with phosphate-buffered saline (PBS), the samples were incubated for 30 min at room temperature with the DNA staining solution containing 30 μg $ml^{-1}$ propidium iodide and 0.5 μg $ml^{-1}$ of RNAase and measured on an Epics Elite

© Cancer Research Campaign 1998

flow cytometer (Coulter Electronics, Hialeah, FL, USA). The DNA histograms were analysed, by the multicycle program (Phoenix Flow Systems, San Diego, CA, USA), for the evaluation of apoptosis and the percentage of cells in the various phases of the cell cycle (Darzynkiewicz et al, 1992). To clarify the kinetics of cell transition through the S and the $G_2$ + M phases of the cell cycle, a simple stathmokinetic (or 'mitotic arrest') experiment (Traganos and Kimmel, 1990) was performed. Before harvesting, the cells were treated with 0.1 μg $ml^{-1}$ of the mitotic blocker colcemid (Gibco, Grand Island, NY, USA) for 8 h. In this way, all those cells which are in late S phase and in $G_2$ phase are arrested in M phase at the time of harvesting. The comparison of the colcemid-induced increase in the $G_2$ + M peaks of exponentially growing untreated and 4-HPR-treated cultures, may reveal perturbations in cell progression through S and $G_2$ cell cycle phases.

### IGF-I/BP complex separation and radioimmunoassay

Aliquots of concentrated CM from treated (5 μM 4-HPR for 48 h) and untreated cells were analysed to determine the concentration of IGF-I-like material using a specific radioimmunoassay (RIA), as previously described (Favoni et al, 1995). As IGF-BPs may interfere in a standard RIA, producing false results, a RIA buffer containing heparin, which allows IGF-I/IGF-BP dissociation, was used (Favoni et al, 1995). In addition, a separate assay for binding proteins was performed to ensure complete BP removal: heparin-treated samples were dried and resuspended in 0.1% BSA–PBS containing 30 000 counts per minute of radiolabelled IGF-I and incubated overnight at 4°C. After incubation for 30 min on ice and centrifugation, unbound labelled IGF-I was precipitated with 0.5 ml of ice-cold activated charcoal (5% w/v suspension in 2% BSA–PBS). Labelled IGF-I which was complexed to binding proteins present in the sample and remained in the supernatant was counted on a Beckman 5500B γ-counter.

### Electrophoresis and Western ligand analysis

Appropriate volumes of concentrated CM were resolved by sodium dodecyl sulphate-polyacrylamide gel electrophoresis (SDS-PAGE) and blotted on nitrocellulose to quali-quantitatively characterize IGF-BPs, using the procedures previously described (Favoni et al, 1994). Binding proteins were visualized by autoradiography and their molecular weight estimated by comparison to defined low-range (18 000–106 000 $M_r$) prestained molecular weight markers (Bio-Rad Laboratories, Richmond, CA, USA). Specific pure human recombinant IGF-binding proteins (hr-IGF-BP4, hr-IGF-BP2, hr-IGF-BP5 from Austral Biologicals, San Ramon, CA, USA; hr-IGF-BP3 from UBI, Lake Placid, NY, USA and hr-IGF-BP1 from Dr GN Cox, Synergen, Boulder, CO, USA) were run as well; their quantities and the degree of variation were calculated by a densitometric analysis of the autoradiography with a LKB Ultroscan XL laser densitometer (Pharmacia/LKB, Uppsala, Sweden).

### Binding experiments

Binding of IGF-I to its specific cell-surface type I IGF receptor (IGF-R) and its modulation by 4-HPR was studied by radioreceptor assay, using the procedure previously described (de Cupis et al, 1995). Briefly, cells were plated in duplicate in 24-well plates at a density of 100 000 to 200 000 cells per well in 1 ml of phenol red-free DMEM supplemented with 5% dextran charcoal–steroid depleted FCS in the presence or absence of 5 μM 4-HPR and incubated under standard conditions. After 48 h, the medium was replaced by binding buffer for one additional hour at 37°C. Competition was carried out by adding 30 000 counts per minute of [$^{125}$I]IGF-I to each well along with increasing concentrations (0.195–400 ng $ml^{-1}$) of competing ligand. At the completion of the binding period (2 h at 4°C with gentle shaking), cells were washed three times with ice-cold washing buffer (0.1% BSA–Hanks' balanced salt solution), after which the monolayers were harvested in lysing buffer. Bound IGF-I was determined on a Beckman 5500B γ-counter and the specific binding was expressed as the percentage of bound IGF-I in the absence of competing peptide. Scatchard analysis was performed to determine the dissociation constant ($K_d$) and the maximum binding capacity ($B_{max}$) relative to 4-HPR-treated and untreated cells. According to some experimental studies (Hsu and Oleksky, 1992; Heding et al, 1996), which report that native IGF-I can also bind to cell surface-associated BPs, a group of assays with Des-(1–3)IGF-I was carried out to obtain a convincing interpretation of the competitive binding data. Des-(1–3)IGF-I is an IGF-I derivative, with glycine, proline and glutamic residues cleaved from the N-terminus, which binds mainly to cell-surface receptors and poorly to the IGF-BPs. In radioreceptor assays, following the same experimental procedure described above, increasing concentrations of Des-(1–3)IGF-I (0.195–400 ng $ml^{-1}$) as the competing ligand rather than IGF-I were used.

### RNA isolation and ribonuclease protection assay

RNA was isolated from cells using the method of Chirgwin et al (1979), and integrity of the total cellular RNA was measured by formaldehyde gel electrophoresis. Ribonuclease (RNAase) protection assay was performed as previously described (Yee et al, 1988). Briefly, 20 μl of total RNA from untreated and 48-h 4-HPR-treated cells (5 μM) was hybridized with radiolabelled probes overnight at 50°C. After ribonuclease A digestion, protected fragments were visualized by denaturing polyacrylamide gel electrophoresis. Probes used in this study were transcribed from a 293-bp *Ava*I–*Ava*I fragment of the IGF-R (Yee et al, 1989, 1992). A probe transcribed from the 36B4 cDNA was used as a loading control. 36B4 encodes for a ribosomal protein that is not regulated by hormones in breast cancer cells (Laborda, 1991). The RNA samples were hybridized simultaneously with both probes. pBR322 digested with *Msp*I were end labelled and used as size markers. The protected fragments were quantified using an Ambis radioanalytical scanner. To determine the levels of type I IGF-receptor mRNA, the net counts for the receptor protected fragment were divided by the net counts for the 36B4 mRNA species.

### Statistical analysis

Statistical significance of the experimental results, reported as a mean percentage ± standard error, was evaluated by the non-parametric Wilcoxon test.

### RESULTS

### Inhibition of growth by 4-HPR

The influence of increasing concentrations of 4-HPR and duration of 4-HPR exposure on breast cancer cell growth is shown in Figure

© Cancer Research Campaign 1998

**Figure 1** Time– and dose–response effects of 4-HPR on proliferation of human breast cancer cells MCF-7 (ER+) and MDA-MB231 (ER–) and the pseudo-normal cell line HBL-100. Points, expressed as a percentage of control, represent the absorbance measured at 540 nm and are the mean of three independent experiments performed in quadruplicate. Standard error ranged from 1 to 6%. ○, 48 h; ●, 72 h; □, 96 h. All the results are significant ($0.005 \leq P \leq 0.009$; $P = 0.034$ only for MCF-7 1 μM 4-HPR at 48 h) except for 1 μM 4-HPR at 48 h for MDA-MB231 and for 1–5 μM 4-HPR at 48, 72 and 96 h for HBL-100

**Figure 2** Cell growth recovery evaluated by MTT colorimetric assay after 48 h of drug treatment (□) followed by 48 h (**A**) and 72 h (**B**) of culture in drug-free medium (■). Columns represent the mean ± SE of three independent experiments: (1) untreated cells (▧); (2) 1 μM 4-HPR; (3) 1 μM 4-HPR + fresh medium; (4) 1.5 μM 4-HPR; (5) 1.5 μM 4-HPR + fresh medium; (6) 2.5 μM 4-HPR; (7) 2.5 μM 4-HPR + fresh medium; (8) 5 μM 4-HPR; (9) 5 μM 4-HPR + fresh medium; (10) 10 μM 4-HPR; (11) 10 μM 4-HPR + fresh medium. Significant effect: $0.005 \leq P \leq 0.01$ (no symbol on the top of the column); °$P = 0.006$; *not significant

1. Growth curves of MTT experiments showed that both MCF-7 and MDA-MB231 breast cancer cell lines are sensitive to 4-HPR in a time- and concentration-dependent manner: the pattern of growth inhibition of the two cell lines was similar (Figure 1). Specifically, cell proliferation was not affected at the dose of 1 μM 4-HPR except for MDA-MB231 at 96 h, whereas it was dramatically inhibited at one log higher concentration. Overall, MCF-7 cells appeared more sensitive to short-term exposure to 4-HPR, whereas MDA-MB231 cells seemed to be most sensitive after a longer exposure. In contrast, the pseudo-normal HBL-100 breast-

© Cancer Research Campaign 1998

**Table 1** Cell cycle distribution of MCF-7, MDA-MB231 and HBL-100 cell lines untreated and treated with 5 μM 4-HPR for 24, 48 and 72 h, stained with propidium iodide and measured by flow cytometry (means ± SE)

| | Cell line | | | | | | | | | | | | | | |
|---|---|---|---|---|---|---|---|---|---|---|---|---|---|---|---|
| | MCF-7 | | | | | MDA-MB231 | | | | | HBL-100 | | | | |
| | $G_1$ (%) | S | $G_2$ + M | $G_2$ + M[a] | Aptoptotic cells (%) | $G_1$ (%) | S | $G_2$ + M | $G_2$ + M[a] | Aptoptotic cells (%) | $G_1$ (%) | S | $G_2$ + M | $G_2$ + M[a] | Aptoptotic cells (%) |
| Untreated | 50 ± 4 | 38 ± 5 | 12 ± 2 | 21 ± 3 | 0 | 49 ± 2 | 38 ± 1 | 13 ± 1 | 36 ± 4 | 0 | 53 ± 4 | 30 ± 4 | 13 + 3 | 31 ± 5 | 0 |
| 4-HPR treated | | | | | | | | | | | | | | | |
| 24 h | 67 ± 2 | 25 ± 2 | 8 ± 1 | 16 ± 3 | 0 | 54 ± 4 | 32 ± 2 | 14 ± 2 | 38 ± 4 | 0 | 59 ± 5 | 26 ± 5 | 15 ± 2 | 26 ± 2 | 0 |
| 48 h | 74 ± 5 | 13 ± 3 | 13 ± 2 | 16 ± 4 | 30–40 | 63 ± 10 | 27 ± 11 | 10 ± 1 | 16 ± 7 | 0–5 | 73 ± 4 | 19 ± 4 | 8 ± 1 | 14 ± 1 | 0 |
| 72 h | 74 ± 1 | 16 ± 2 | 10 ± 2 | 9 ± 2 | 60–80 | 66 ± 1 | 25 ± 3 | 9 ± 2 | 9 ± 2 | 20–30 | 69 ± 1 | 23 ± 1 | 8 ± 1 | 11 ± 1 | 0 |

[a]Cells exposed to 0.1 μg ml$^{-1}$ colcemid for 8 h before harvesting.

**Figure 3** DNA content distribution in (**A**) untreated MCF-7 cells and MCF-7 cells treated with 5 μM 4-HPR for (**B**) 48 h or (**C**) 72 h. Histograms are representative of two independent experiments

**Figure 4** Inhibition of exogenous IGF-I-stimulated cell proliferation induced by simultaneous 4-HPR treatment for 24 h on MCF-7 cell line. Bars, expressed as a percentage of control, represent the [$^3$H]dThd incorporated into DNA. Standard error ranged from 6 to 10%. Values, representing the mean of four independent experiments performed in triplicate, are significant ($P$ < 0.001). ■, Control; □, IGF-I 10 nM; ▧, 4-HPR 1 μM; ▨, 4-HPR 5 μM; ▥, IGF-I + 4-HPR 1 μM; ▩, IGF-I + 4-HPR 5 μM

derived cell line was unaffected at doses below 5 μM (Figure 1). Similar results were obtained with thymidine incorporation experiments, in which 4-HPR (range 1–10 μM) induced a significant progressive inhibition of [$^3$H]dThd uptake (26–99%), with an $IC_{50}$ of approximately 2.5 μM and 100% growth arrest within 24 h. Again, the inhibitory activity of 4-HPR on HBL-100 was less evident both at the lowest and the highest retinoid concentrations (data not shown).

To establish whether the antiproliferative effect of 4-HPR (1–10 μM 4-HPR for 48 h) was reversible upon its removal, MTT experiments of cell growth recovery were performed. Upon drug withdrawal, MCF-7 and MDA-MB231 cell lines again acquired proliferative activity by 48 and 72 h in a 4-HPR-free culture medium at all concentrations except for 10 μM (recovery range 4–74% and 4–35% compared with 4-HPR-treated cells for MCF-7 and MDA-MB231 respectively), indicating that part of the inhibitory effect of 4-HPR was cytostatic rather than cytotoxic. As expected, growth recovery of both cancer cell lines tended to decrease with increasing 4-HPR concentrations (Figure 2).

*© Cancer Research Campaign 1998*

**Figure 5** Effect of 5 µM 4-HPR on the secretion of immunoreactive IGF-I-like material into the CM from MCF-7, MDA-MB231 and HBL-100 cell lines. Data are reported as the means ± SE of four independent experiments performed in duplicate. □, Control; ■, 4-HPR-treated. Statistical significance: $P = 0.006$, except for HBL-100

**Figure 6** (**A**) Ligand blot of CM obtained from MCF-7 and MDA-MB231 human cells in the absence or presence of 5 µM 4-HPR. Molecular weight standard is represented on the left axis, the molecular weight of IGF-BPs is indicated on the right axis. Lanes: 1, MCF-7 untreated; 2, MCF-7 4-HPR-treated; 3, MDA-MB231 untreated; 4, MDA-MB231 4-HPR-treated; A, hr-IGF-BP4; B, hr-IGF-BP1; C, hr-IGF-BP5; D, hr-IGF-BP2; E, hr-IGF-BP3, 300 ng per each IGF-BP were run. (**B**) Amounts of IGF-BP4, 1, 5 and 2 secreted by MCF-7 and MDA-MB231 cell lines at the steady-state level (□) and after 4-HPR treatment (■). Columns represent the area (mm$^2$) of each binding protein, expressed in arbitrary units, obtained with densitometric analysis of the autoradiography

### DNA content analysis

Appearance of fractional DNA content, typical of apoptotic cells (Darzynkiewicz et al, 1992), was found in 30–40% and in 60–80% of MCF-7 cells following 48 and 72 h of exposure to 5 µM 4-HPR respectively (Table 1 and Figure 3). Moreover, an accumulation of MCF-7 cells in the $G_1$ phase was already detectable at 24 h (Table 1). The 'mitotic arrest' assay showed that after 24 h 4-HPR exposure, the colcemid-induced increase in the $G_2$ + M percentage was similar to that observed in the untreated cells. In contrast, in cells exposed for 48 and 72 h to 4-HPR, treatment with colcemid did not modify the $G_2$ + M percentage, indicating that cell progression through S- and $G_2$ phases was strongly curbed. In the ER– MDA-MB231 cells, only a few apoptotic cells (0–5%) were evident at 48 h, rising to 20–30% after 72 h of retinoid exposure. An increase in the $G_1$ population was observed after 48 and 72 h, and progression through S- and $G_2$ phases was strongly delayed after 48 and 72 h treatment (Table 1). In contrast, no apoptotic cells were found in 4-HPR-treated HBL-100 cells. There was a moderate increase in the $G_1$ phase fraction, while progression through S- and $G_2$ phases was not affected (Table 1).

### Modulation of the IGF-I system by 4-HPR

To assess whether the inhibition of cell growth induced by 4-HPR was directly mediated by down-regulation of the growth factor mitogenic activity, exogenous hr-IGF-I-stimulated cell proliferation was measured by [$^3$H]dThd incorporation assay during concurrent administration of 1 or 5 µM 4-HPR. Exposure to 10 nM hr-IGF-I induced cell growth stimulation only in MCF-7 cells (68%) after 24 h ($P < 0.001$). This effect was abolished by 1 µM and even counteracted by 5 µM 4-HPR, reaching a 46% growth inhibition from baseline (Figure 4).

The presence of immunoreactive IGF-I-like material in the CM of all three cell lines was demonstrated by quadruplicate radioimmunoassay experiments shown in Figure 5. As expected, higher amounts of secreted polypeptide were found in ER– MDA-MB231 than in ER+ MCF-7 cell lines (in our experiment more than sixfold). Treatment with 5 µM 4-HPR for 48 h induced a 38% and 90% reduction in the growth factor concentration in the CM from MCF-7 and MDA-MB231 cells respectively. In contrast, the synthetic retinoid had a marginal effect on the secretion of IGF-I-like material in HBL-100 (Figure 5).

The presence of IGF-BP molecular species of 24 000, ~32 000 and 34 000 $M_r$, corresponding to IGF-BP4, IGF-BP5 and IGF-BP2, was detected under basal culture conditions in the CM from MCF-7 cells (Figure 6A). The MDA-MB231 cell line also synthesized and secreted IGF-BP4 together with IGF-BP1 (30 000 $M_r$). IGF-BP3, the largest 42–46 000 $M_r$ IGF-BP, was also identified, albeit very faintly, in MDA-MB231 cells, while it was not expressed in MCF-7 cells (Figure 6A). Treatment with 4-HPR induced a remarkable reduction in the amount of IGF-BP4 (by 67%) and IGF-BP5 (by 87%) and a 16% reduction in the amount of IGF-BP2 secreted in the CM derived from MCF-7 cells (Figure 6B). In the CM collected from MDA-MB231, both IGF-BP4 and IGF-BP1 were decreased, by 24% and 20% respectively, while IGF-BP3, which was very poorly expressed, appeared to be reduced as well, although its levels were undetectable by densitometric analysis (Figure 6B). The analysis of CM from HBL-100 cells did not reveal any appreciable presence of IGF-BPs (data not shown).

© Cancer Research Campaign 1998

**Figure 7** (**A**) Radioreceptor assays performed with either IGF-I (●) and Des-(1–3)IGF-I (△) as competitor ligands, used at concentrations of 0.195–400 ng ml⁻¹. Data represent the means ± SE of three independent experiments performed in duplicate. (**B**) Scatchard analysis of competitive binding data using two wells per point in three experiments. Bound/free ratios vs specific bound are indicated □, control; ■, 4-HPR-treated. Insets: values of $K_d$ (nM), $B_{max}$ (pM), and number of high-affinity sites (× $10^4$) per cell for untreated (control) and 4-HPR-treated cells

The ability of 4-HPR to modify IGF-I binding to MCF-7, MDA-MB231 and HBL-100 cell-surface IGF-R was assessed by competitive binding assay and Scatchard analysis of the data. Experiments carried out in the presence of either hr-IGF-I or its truncated analogue Des-(1–3)IGF-I showed basically no differences in the polypeptides' binding ability because the same amount (≈ 80%) of the total cell-associated [$^{125}$I]-IGF-I was displaced by both native and Des-(1–3)IGF-I in cell lines under study (Figure 7A; HBL-100 data not shown). On the basis of these results, subsequent radioreceptor assays were performed in accordance with the standard procedure, only with the physiological species of IGF-I. Both MCF-7 and MDA-MB231 cells had approximately 90 000 sites per cell (Figure 7B, inset), whereas HBL-100 had about 60 000 sites per cell. The $K_d$ was in the nanomolar range for all three cell lines (data not shown). Scatchard analysis revealed a linear plot, suggesting a typical (de Cupis et al, 1995; Hodgson et al, 1995) single binding site for IGF-I (Figure 7B). The maximal binding capacity was about 40 pM for both breast cancer cells and the pseudo-normal cell line. Forty-eight hours' pretreatment with 5 μM 4-HPR did not significantly change the binding affinity, but reduced the number of cell-surface receptors in MCF-7 (by 30%) and MDA-MB231 (by 12%). In addition, the $B_{max}$ values for MCF-7 and MDA-MB231 were reduced by about 60% and 50% respectively (Figure 7B, inset). In contrast, IGF-I receptor number, $K_d$ and $B_{max}$ of HBL-100 were only marginally reduced by 4-HPR (data not shown).

Finally, treatment with 4-HPR at 5 μM for 48 h induced a decrease in steady-state levels of type I IGF-receptor mRNA by 30% and 37% of control in MCF-7 and MDA-MB231 cells respectively (Figure 8).

### DISCUSSION

Inhibition of the IGF-I system, the most potent mitogen for breast cancer cells, is an important mechanism by which natural retinoids antagonize in vitro breast cancer cell growth (Fontana et al, 1991; Adamo et al, 1992; Roman et al, 1992; van der Burg et al, 1993; Rubin et al, 1994). Consensus, moreover, points to the dependence of these retinoids' growth-inhibitory effect on the expression of the ER pathway (van der Burg et al, 1993; Roman et al, 1992;

© Cancer Research Campaign 1998

**Figure 8** (**A**) RNAase protection analysis of IGF-R gene expression. Sizes of IGF-R and 36B4 cDNA are marked on the right vertical axis. Lanes: 1, markers; 2, probe transcribed from 36B4 cDNA; 3, MCF-7 USA as control; 4, MCF-7 untreated; 5, MCF-7 4-HPR-treated; 6, MDA-MB231 untreated; 7, MDA-MB231 4-HPR-treated. (**B**) Quantification of protected fragments obtained by an Ambis radioanalytic scanner. □, Control; ■, 4-HPR-treated

Rubin et al, 1994). Indeed, ER– breast cancer cells do not respond to RA and 9-*cis*-RA, and IGF-R expression is not down-regulated by these compounds in MDA-MB231 cells (Rubin et al, 1994). 4-HPR is a well-tolerated synthetic retinoid which is currently being evaluated by our group in a large prevention trial of second primary breast cancer (Costa et al, 1994). Within the context of the clinical study, we observed a significant inhibition of plasma IGF-I levels in women receiving 4-HPR (Torrisi et al, 1993). To provide further insight into this modulatory activity, we studied the in vitro effect of 4-HPR on the IGF-I system and whether this action was related to cell growth inhibition.

Our data indicate that 4-HPR can significantly inhibit the growth of both ER+ MCF-7 and ER– MDA-MB231 breast cancer cell lines. Although the pattern of growth inhibition varied depending on the cell line in a time- and concentration-dependent manner, the finding that the MDA-MB231 cell line was sensitive to 4-HPR differs from what is commonly observed with RA and 9-*cis*-RA (Roman et al, 1992; van der Burg et al, 1993; Rubin et al, 1994). It is, however, consistent with a recent observation by Sheikh et al (1995), who showed that 1 μM 4-HPR was more potent than RA, at the same concentration, as antiproliferative agent on both MCF-7 and MDA-MB231 cell lines and that it inhibited the growth of RA-resistant cells (Sheikh et al, 1995). Although in our experiments cell lines appeared less sensitive to that dose, the observed inhibition of ER– cells seemingly supports the contention that at least part of the mechanism of 4-HPR activity is not mediated by binding to retinoid receptors (Delia et al, 1995; Lotan, 1995; Sheikh et al, 1995).

Cell exposure to the synthetic retinoid led to perturbations in cell cycle progression in both neoplastic cell lines, as demonstrated by flow cytometric studies. However, induction of apoptosis was more rapid and extensive in MCF-7 than in MDA-MB231 cells. Moreover, apoptosis was achieved at 5 μM or higher doses, while below that threshold the growth-inhibitory effect was reversible. Again, the preferential sensitivity to apoptosis in MCF-7 in comparison with MDA-MB231 is consistent with the results by Sheikh et al (1995), although these authors observed apoptosis at 4-HPR doses as low as 1 μM. Thus, taken together, these data suggest a link between the induction of apoptosis by the retinoid and the ER pathway. In contrast to the data of Sheikh et al (1995), Fanjul et al (1996) have recently shown that 4-HPR is a selective ligand of retinoid receptors. In their experiments, 4-HPR was able to transactivate moderately with RARγ and, to a lesser extent, with RARβ in comparison with RA. More importantly, however, 4-HPR was found to efficiently transrepress the AP-1 complex with RARα, induce apoptosis in different systems and, finally, overexpress RARγ. Although these effects could be detectable starting from 5 μM, they were completely evident at 10–20 μM (Fanjul et al, 1996). While this dose range exceeds the dose currently employed in the clinic and is likely to be accompanied by an excessive toxicity in humans, it is not known whether the prolonged exposure to the drug in prevention trials might compensate for this dose threshold effect. It is also unclear whether the apparent discrepancies between these studies are traceable to the presence of two distinct mechanistic pathways, one operating at lower doses, independent of retinoid receptors, and the other acting at higher doses through the binding to nuclear receptors.

Our results indicate that 4-HPR significantly inhibits the IGF-I system and that this modulatory activity mediates the antiproliferative effect of the retinoid. In fact, concomitant administration of 4-HPR was able to counteract the proliferative stimulation induced by exogenous IGF-I. As expected, ER– cells constitutionally secrete higher concentrations of IGF-I-like material (Huff et al, 1986), and this partly explains the relative higher inhibition of its secretion by 4-HPR in MDA-MB231 compared with MCF-7 cells. Although breast cancer cells do not synthesize IGF-I mRNA (Yee et al, 1989), a closely related peptide could be detected in all cell lines using an anti-IGF-I polyclonal antibody. Moreover, binding protein assays were performed after dissociation and separation of the whole binding protein component, in order to exclude a possible anti-IGF-I antibody/IGF-BPs cross-reactivity. Finally, IGF-II RNAase protection assay showed no significant IGF-II mRNA expression in the cancer cell line examined (data not shown). Taken together, these results indicate that the RIA quantified a molecule closely related to IGF-I.

Our experiments also show that 4-HPR was able to down-regulate IGF-I binding to IGF-R significantly in both tumour cell lines. This down-regulation appears to be due to the inhibition of IGF-R gene expression. These data are noteworthy, in as much as recent studies have shown that neither RA nor 9-*cis*-RA down-regulate the expression of type I IGF receptor in MDA-MB231 cells (Rubin et al, 1994). Given the pivotal involvement of this receptor in the control of proliferation, apoptosis and carcinogenesis (Baserga, 1995), these effects are likely to be important in the preventive activity of 4-HPR. Administration of 4-HPR caused a

© Cancer Research Campaign 1998

down-regulation in all IGF-BPs secreted by both cell lines, with expected differences in secretion values between ER+ and ER– cells (Figueroa and Yee, 1992). Since IGF-BP4, -BP5 and -BP2 have been shown to be positively involved in controlling cellular proliferation (Figueroa and Yee, 1992), the retinoid-induced cell growth inhibition may also be related to the observed decrease in their secretion. Indeed, in a recent study, low IGF-BP4 was correlated with improved survival in women with large tumour size (Yee et al, 1994). Secretion of IGF-BP1 was evident only in MDA-MB231, as previously described (Yee et al, 1991). Because the in vitro biological effect of IGF-BP1 tends to vary according to experimental systems (Fugueroa and Yee, 1992; McGuire et al, 1992), its mild down-regulation by 4-HPR does not lend itself to easy interpretation.

Consistent with most previous findings (Clemmons et al, 1990; Figueroa et al, 1993), the presence of IGF-BP3 was not identifiable in MCF-7, whereas it was only slightly detectable in untreated MDA-MB231 cells. There is also evidence that IGF-BP3 is under the control of several ligands of the steroid superfamily. For instance, its appearance in CM from MCF-7 and MDA-MB231 has been documented after RA treatment (Adamo et al, 1992; Sheikh et al, 1993; Gucev et al, 1996), and expression of gene and protein is enhanced by anti-oestrogen and decreased by oestrogen administration (Huynh et al, 1996). In our hands, exposure to 4-HPR was associated with undetectable secretion in MCF-7 and with a minimal decrease in IGF-BP3 in MDA-MB231. Differences in techniques, in the compounds used and the heterogeneity of cell cultures may account for these discrepancies. Indeed, we have previously reported on the low detectability of IGF-BP3 in the CM from MDA-MB231 cells (de Cupis et al, 1995).

In conclusion, our results indicate that 4-HPR inhibits the growth of both MCF-7 and MDA-MB231 human breast cancer cell lines. These effects are associated and, at least in part, mediated by a significant down-regulation of the IGF-I system and are likely to characterize the clinical preventive potential of this retinoid. Another study, extended to a wider panel of ER+ and ER– breast cancer cell lines, is being planned in order to obtain further evidence which will allow extrapolation of these preliminary findings to all breast cancers and lines.

### ACKNOWLEDGEMENTS

The authors would like to thank Mr Tom Wiley for the revision of the text. A de Cupis is supported by a grant from Associazione Italiana per la Ricerca sul Cancro (AIRC).

### REFERENCES

Adamo ML, Shao ZM, Lanau F, Chen CC, Clemmons DR, Roberts Jr CT, LeRoith D and Fontana JA (1992) Insulin-like growth factor-I (IGF-I) and retinoic acid modulation of IGF-binding protein (IGF-BPs): IGF-BP-2, -3, -4 gene expression and protein secretion in a breast cancer cell line. *Endocrinology* **131**: 1858–1866

Baserga R (1995) The insulin-like growth factors I receptor: a key to tumor growth? *Cancer Res* **55**: 249–252

Bruning PF, Bonfrer JMG, van Noord PAH, Hart AAM, de Jong-Bakker M and Nooijen WJ (1992) Insulin resistance and breast cancer risk. *Int J Cancer* **52**: 511–516

Chirgwin JM, Przybyla AI, MacDonald RJ and Rutter WJ (1979) Isolation of biologically active ribonucleic acid from sources enriched in ribonucleases. *Biochemistry* **18**: 5294–5299

Clemmons DR, Camacho-Hubner C, Coronado E and Osborne CK (1990) Insulin-like growth factor binding protein secretion by breast carcinoma cell lines: correlation with estrogen receptor status. *Endocrinology* **127**: 2679–2686

Costa A, Formelli F, Chiesa F, Decensi A, De Palo G and Veronesi U (1994) Prospects of chemoprevention of human cancers with the synthetic retinoid fenretinide. *Cancer Res* **54** (suppl.): 2032–2037

Darzynkiewicz Z, Bruno S and Del Bino G (1992) Features of apoptotic cells measured by flow cytometry. *Cytometry* **13**: 795–808

Decensi A, Costa A, De Palo G, Formelli F, Marubini E, Mariani L, Fontana V and Veronesi U (1997) Retinoid–menopause interactions in a breast cancer prevention trial (abstract 3548). *Proc Am Assoc Cancer Res* **38**: 529

de Cupis A, Noonan D, Pirani P, Ferrera A, Clerico L and Favoni RE (1995) Comparison between novel steroidal-like and conventional non-steroidal antiestrogen in inhibiting estradiol- and IGF-I-induced proliferation of human breast cancer-derived cell lines. *Br J Pharmacol* **116**: 2391–2400

Delia D, Di Aiello A, Formelli F, Fontanella E, Costa A, Miyashita T, Reed JC and Pierotti MA (1995) Regulation of apoptosis induced by the retinoid N-(4-hydroxyphenyl)retinamide and effect of deregulated bcl-2. *Blood* **85**: 359–367

Evans RM (1988) The steroid and thyroid hormone receptor superfamily. *Science* **240**: 889–895

Fanjul AN, Delia D, Pierotti D, Rideout D, Qiu J and Pfahl M (1996) 4-hydroxyphenylretinamide is a highly selective activator of retinoid receptors. *J Biol Chem* **271**: 22441–22446

Favoni RE, de Cupis A, Ravera F, Cantoni C, Pirani P, Ardizzoni A, Noonan D and Biassoni R (1994) Expression and function of the insulin-like growth factor-I system in human non-small cell lung cancer and normal lung cell lines. *Int J Cancer* **56**: 858–866

Favoni RE, de Cupis A, Perrotta A, Sforzini S, Amoroso D, Pensa F and Miglietta L (1995) IGF-I and IGF-binding proteins blood serum levels in women with early- and late-stage breast cancer: mutual relationship and possible correlations with patients hormonal status. *J Cancer Res Clin Oncol* **121**: 674–682

Figueroa JA and Yee D (1992) The insulin-like growth factor binding proteins (IGF-BPs) in human breast cancer. *Breast Cancer Res Treat* **22**: 81–90

Figueroa JA, Jackson J, McGuire WL, Krywicki RF and Yee D (1993) Expression of insulin-like growth factor binding proteins in human breast cancer correlates with estrogen receptor status. *J Cell Biochem* **52**: 196–205

Fontana JA, Burrows-Mezu A, Clemmons DR and LeRoith D (1991) Retinoid modulation of estradiol-stimulated growth factor binding proteins and inhibition of breast carcinoma proliferation. *Endocrinology* **128**: 1115–1122

Gucev ZS, Youngman O, Kelley KM and Rosenfeld RG (1996) Insulin-like growth factor binding protein 3 mediates retinoic acid- and transforming growth factor b2-induced growth inhibition in human breast cancer cells. *Cancer Res* **56**: 1545–1550

Heding A, Gill R, Ogawa Y, De Meyts P and Symoko ZM (1996) Biosensor measurement of the binding of insulin-like growth factors I and II and their analogues to the insulin-like growth factor-binding protein-3. *J Biol Chem* **271**: 13948–13952

Hodgson DR, May FEB and Westley BR (1995) Mutations at positions 11 and 60 of insulin-like growth factor 1 reveal differences between its interactions with the type I insulin-like growth factor receptor and the insulin receptor. *Eur J Biochem* **233**: 299–309

Hsu D and Oleksky JM (1992) Characterization of insulin-like growth factor (IGF) binding proteins and their role in modulating IGF-I action in BHK cells. *J Biol Chem* **267**: 25576–25582

Huff KK, Kaufman D, Gabbay KH, Spencer EM, Lippman ME and Dickson RB (1986) Secretion of an insulin-like growth factor-I-related protein by human breast cancer cells. *Cancer Res* **46**: 4613–4619

Huynh HM, Yang X and Pollack M (1996) Estradiol and antiestrogens regulate a growth inhibitory insulin-like growth factor binding protein 3 autocrine loop in human breast cancer cells. *J Biol Chem* **271**: 1016–1021

Kazer RR (1995) Insulin resistance, insulin-like growth factor I and breast cancer: a hypothesis. *Int J Cancer* **62**: 403–406

Laborda J (1991) 36B4 cDNA used as an estradiol-independent mRNA control is the cDNA for human acidic ribosomal phosphoprotein PO. *Nucleic Acids Res* **19**: 3998

Lacroix A and Lippman ME (1980) Binding of retinoids to human breast cancer cell lines and their effects on cell growth. *J Clin Invest* **65**: 586–591

Leid M, Kastner P and Chambon P (1992) Multiplicity generates diversity in the retinoic acid signalling pathways. *Trends Biochem Sci* **17**: 427–433

LeRoith D, Clemmons D, Nissley P and Rechler MM (1992) NIH Conference: insulin-like growth factor in health and disease. *Ann Intern Med* **116**: 854–862

© *Cancer Research Campaign 1998*

LeRoith D, Baserga R, Helman L and Roberts Jr CT (1995) NIH Conference: insulin-like growth factors and cancer. *Ann Intern Med* **122**: 54–59

Li XS, Chen JC, Sheikh MS, Hao ZM and Fontana JA (1994) Retinoic acid inhibition of insulin-like growth factor I stimulation of c-fos mRNA levels in a breast carcinoma cell line. *Exp Cell Res* **211**: 68–73

Lotan R (1979) Different susceptibilities of human melanoma and breast carcinoma cell lines to retinoic acid-induced growth inhibition. *Cancer Res* **39**: 1014–1019

Lotan R (1995) Retinoids and apoptosis: implications for cancer chemoprevention and therapy. *J Natl Cancer Inst* **87**: 1655–1657

Marth C, Bock G and Daxenbichler G (1985) Effect of 4-hydroxyphenylretinamide and retinoic acid on proliferation and cell cycle of cultured human breast cancer cells. *J Natl Cancer Inst* **75**: 871–875

McGuire Jr WL, Jackson JG, Figueroa JA, Shimasaki S, Powell DR and Yee D (1992) Regulation of insulin-like growth factor-binding protein (IGF-BP) expression by breast cancer cells: use of IGF-BP-1 as an inhibitor of insulin-like growth factor action. *J Natl Cancer Inst* **84**: 1336–1341

Moon RC, Thompson HJ, Becci PJ, Grubbs CH, Gander RJ, Newton DL, Smith JM, Philips SL, Henderson WR, Mullen LT, Brown CC and Sporn M (1979) N-(4-hydroxyphenyl)retinamide, a new retinoid for prevention of breast cancer in the rat. *Cancer Res* **39**: 1339–1346

Polanowski FP, Gaffney EV and Burke RE (1976) HBL-100, a cell line established from human breast milk. *In Vitro* **12**: 328

Ponzoni M, Bocca P, Chiesa V, Decensi A, Pistoia V, Reffaghello L, Rozzo C and Montaldo PG (1995) Differential effects of N-(4-hydroxyphenyl)retinamide and retinoic acid on neuroblastoma cells: apoptosis versus differentiation. *Cancer Res* **55**: 853–861

Rishi AK, Shao ZM, Baumann RG, Li XS, Sheikh MS, Kimura S, Bashilerashi N and Fontana JA (1995) Estradiol regulation of the human retinoic acid receptor: a gene in human breast carcinoma cells is mediated via an imperfect half-palindromic estrogen response element and Sp 1 motifs. *Cancer Res* **55**: 4999–5006

Roman SD, Clarke CL, Hall RE, Alexander IE and Sutherland RL (1992) Expression and regulation of retinoic acid receptors in human breast cancer cells. *Cancer Res* **52**: 2236–2242

Rubin M, Fenig E, Rosenauer A, Mendez-Botet C, Achkar C, Bentel JM, Yahalom J, Mendelsohn J and Miller Jr WH (1994) 9-cis retinoic acid inhibits growth of breast cancer cells and down-regulates estrogen receptor RNA and protein. *Cancer Res* **54**: 6549–6556

Schule R, Rangarajan P, Yang N, Kliever S, Ransone LJ, Bolado J, Verma IM and Evans RM (1991) Retinoic acid is a negative regulator of AP-1-responsive genes. *Proc Natl Acad Sci USA* **88**: 6092–6096

Sheikh MS, Shao ZM, Hussain A, Clemmons DR, Chen JC, Roberts Jr CT, LeRoith D and Fontana JA (1993) Regulation of insulin-like growth factor-binding-protein-1, -2, -3, -4, -5, and -6: synthesis, secretion, and gene expression in estrogen receptor-negative human breast carcinoma cells. *J Cell Physiol* **155**: 556–567

Sheikh MS, Shao ZM, Li XS, Ordonez JV, Conley BA, Wu S, Dawson MI, Han QX, Chao WR, Quick T, Niles RM and Fontana JA (1995) N-(4-hydroxyphenyl)-retinamide (4-HPR)-mediated biological actions involve retinoid receptor-independent pathways in human breast carcinoma. *Carcinogenesis* **16**: 2477–2486

Smith MA, Parkinson DR, Cheson BD and Friedman MA (1992) Retinoids in cancer therapy. *J Clin Oncol* **10**: 839–864

Stoll BA (1993) The growth hormone/insulin-like growth factor axis and breast cancer risk. *Breast* **1**: 130–133

Torrisi R, Pensa F, Orengo MA, Catsafados E, Ponzani P, Boccardo F, Costa A and Decensi A (1993) The synthetic retinoid fenretinide lowers plasma IGF-I levels in breast cancer patients. *Cancer Res* **53**: 4769–4771

Traganos F and Kimmel M (1990) The statmokinetik experiment: a single parameter and multiparameter flow cytometric analysis. In *Methods in Cell Biology*, *Flow Cytometry*, Darzynkiewicz Z and Crissman HA (eds), pp. 249–270. Academic Press: New York van der Burg B, Isbrucker L, van Selm-Miltenburg AJP, de Laat SW and van Zoelen EJJ (1990) Role of estrogen-induced insulin-like growth factors in the proliferation of human breast cancer cells. *Cancer Res* **50**: 7770–7774

van der Burg B, van der Leede B, Kwakkenbos-Isbrucker L, Salverda S, de Laat SW and van der Saag PT (1993) Retinoic acid resistance of estradiol-independent breast cancer cells coincides with diminished retinoic acid receptor function. *Mol Cell Endocrinol* **91**: 149–157

Yee D, Cullen KJ, Paik S, Perdue JF, Hampton B, Schwartz A, Lippman ME and Rosen N (1988) Insulin-like growth factor-II mRNA expression in human breast cancer. *Cancer Res* **48**: 6691–6696

Yee D, Paik S, Lebovic GS, Marcus RR, Favoni RE, Cullen KJ, Lippman ME and Rosen N (1989) Analysis of insulin-like growth factor I gene expression in malignancy: evidence for a paracrine role in human breast cancer. *Mol Endocrinol* **3**: 509–517

Yee D, Favoni RE, Lippman ME and Powell DR (1991) Identification of insulin-like growth factor binding proteins in breast cancer cells. *Breast Cancer Res Treat* **18**: 3–10

Yee D, Lebovic GS, Marcus RR and Rosen N (1992) Identification of an alternate type I insulin-like growth factor receptor beta subunit mRNA transcript. *J Biol Chem* **264**: 21439–21441

Yee D, Sjarma J and Hilsenbeck SG (1994) Prognostic significance of insulin-like growth factor-binding protein expression in axillary lymphnode-negative breast cancer. *J Natl Cancer Inst* **86**: 1785–1789

© Cancer Research Campaign 1998